# Mental Health Stigma Among Black Immigrant Women in An Urban Setting

**Published:** 2021

**Authors:** Aderonke Bamgbose Pederson, Valerie Earnshaw, Crystal T. Clark, Katelyn Zumpf, Inger Burnett-Zeigler

**Affiliations:** 1 Department of Psychiatry and Behavioral Sciences, Feinberg School of Medicine, Northwestern University, United Sates; 2 Department of Human Development and Family Sciences, University of Delaware, United Sates

**Keywords:** Mental health, Stigma, Immigrants, Black persons, Religion, Spirituality

## Abstract

**Background:**

Mental health stigma results in unmet mental health needs. Research describing predictors of stigma remains limited among Black immigrants. We aim to examine stigma associated with mental illness among a group of Black immigrant women.

**Methods:**

We examined data from 22 women from two Black immigrant community centers. We collected surveys on demographics, cultural beliefs, migration status, religiosity and mental health stigma. Simple linear regression was used to model the unadjusted association between each component variable and overall stigma scores. All analyses were conducted using R and assumed a two-sided, 5% level of significance.

**Results:**

A linear relationship was found between author-generated scale, the Stigma and Culture Survey (SCS) and the Depression Self Stigma Scale (DSSS). Among respondents, use of religious resources was associated with less stigma (p-value: 0.04). Whereas spirituality and morality was associated with greater stigma (p-value: 0.003). United States citizenship was associated with less stigma (p-value: 0.0001).

**Discussion/Conclusion:**

Religion and spirituality are critical to understanding mental health stigma among Black immigrants. Studies aimed at assessing and reducing stigma need to critically engage with cultural and religious factors.

## Introduction

Black immigrants in the United States are a substantial and growing segment of the United States population who experience significant health disparities but are largely overlooked by research. There are 4.3 million Black immigrants in the United States, majority of Black immigrants have their countries of origin in African and Afro-Caribbean countries. One in ten Black people are foreign born and one in five Black people are either first- or second-generation immigrants^1^. Over the last two decades, the number of Black immigrants in the United States rose 71%, with most coming from Africa^1^. One-third of immigrant families are considered low income and 23% are without a high school diploma^2^. The interaction of race, income and immigration status, among other social determinants of health, places Black immigrants at high-risk for poor physical and mental health outcomes, yet they are the least studied of all immigrant groups in research^3,4^. The NIH has called for more research focusing on how immigrant health may be impacted by stigma^2^.

Stigma, which refers to a “social process involving labeling, stereotyping, separation, status loss, and discrimination in a power context”^5^ and has been identified as a “fundamental cause of health inequities” (Hatzenbuehler et al., 2013), has been recognized as a barrier to mental health treatment and likely plays a significant role among Black immigrants. Evidence suggests that stigma results in lower utilization of mental health treatment and increased severity of mental illness among racial and ethnic minorities^7,8^. Black immigrants tend to have low levels of mental health utilization despite high levels of need^9^. Mental illness is often missed in routine primary care visits, and mental health care utilization is low among Black immigrants^9,10^. In one study, Black immigrants from Ethiopia who did seek care were more likely to consult with traditional healers than health care professionals^11^.

Stigma experienced by Black immigrants may be understudied, in part, because research typically addresses the Black community as a homogenous entity despite the differences in culture and background^12^. Cultural beliefs and practices often intersect with stigma and impact mental health among Black immigrants^9^. For example, among Black immigrants, culture, religion, spiritual beliefs and language variance influence health care experiences^4^. Often when mental health stigma is addressed, too little attention is paid to the cultural context and background of the population studied. Existing stigma assessment scales do not address culturally specific beliefs and are validated within Western contexts^13^. This homogenous approach towards Black people does not take into account how immigrant experiences undermines our ability to understand stigma, which tends to be rooted in culture-specific beliefs^12–15^.

Research on health inequalities among the Black population tends to focus on African Americans whose families have been in the United States for many generations^3,12^. There is often an exclusion of African immigrants, those born in Africa, overlooking how immigrant backgrounds may affect health outcomes^3,12^. Beyond immigrants in the U.S, we also have a conspicuous absence of mental health research from low to middle-income countries^16^, where many Black immigrants have their origins. In this exploratory pilot study we focus on the experiences of Black immigrants from two Black immigrant community based organizations in an urban setting to understand the role of mental illness stigma among a population of Black immigrant women, a population with shared experiences.

We focus on perceived and experienced stigma: experienced stigma refers to the experience of negative attitudes, beliefs or discrimination that one may experience from others; perceived stigma refers to the perception of societal beliefs, attitudes and discrimination held by the community towards persons with mental illness^17,18^. We explored whether preference for resources (such as religious or community resources) was associated with stigma. In addition to using a standardized and validated scale to measure stigma, we also created a measure to address culture specific beliefs related to stigma. We hypothesize that demographic factors including immigrant status, culture specific beliefs and religious views may influence stigmatizing views of mental illness and mental health treatment.

## Methods

### Recruitment

Study activities took place at the Pan African Association (PAA) in the Edgewater neighborhood of Chicago, Illinois and at the United African Organization (UAO) located in the Bronzeville neighborhood on the south side of Chicago, Illinois. Both organizations serve Black immigrants in an urban setting. The PAA serves primarily North and East Africans while the UAO serves primarily West Africans. Black immigrants from Africa may share similar experiences in migrating to the United States. Each organization serves several hundred Black immigrants each year in a variety of services including in immigration, employment, health resources and relocation support. Flyers, posters and bulletins were placed in public announcement spaces in the offices of these community organizations. The staff at both organizations shared announcements with members using flyers and brochures. Flyers described the study, seeking Black immigrant women who have ever experienced stress, anxiety or low mood were distributed.

### Ethics review

The study was approved by the institutional review board at Northwestern University.

### Inclusion criteria

Among individuals in the community who are affiliated with the PAA and UAO community organizations, eligible participants responded to flyers and posters and they were invited to complete questionnaires. Persons who were women, between ages 18–65 years old and English speaking, were eligible to participate (N = 22). Within African communities, discussion around mental health topics may be challenging in mixed gender groups, therefore we kept groups specific to women to reduce burden of disclosure of personal views on mental health. Non-English speaking participants were excluded from the study. All participants had an affiliation to the community-based organizations.

## Measures

We explore the content and characteristics of mental illness stigma and its relationship to demographic factors (i.e., age, marital status, US citizenship and socioeconomic status) among Black immigrant women. We created an original scale (The Stigma and Culture Survey) to capture culture specific mental health conceptualization and experiences that may result in stigmatizing views evident in the literature and compared it to a validated stigma scale (Depression Self Stigma Scale). Our survey assessed views on suicidality, depression, spirituality and morality around mental health. Current validated mental health stigma scales do not address specific cultural and religious views that may influence health experiences for Black immigrants.

### The following measures were administered:

1. A socio-demographic questionnaire that included the following variables: age group, marital status, gender, United States citizenship, education, employment status, socio-economic status as well as health insurance status.
2. The Stigma and Culture Survey, is a self-report stigma survey created by the first author (A.P.) to assess views on prayer, spirituality, preferred sources for support for mental illness. Sample questions included views that mental illness is caused by evil spirits or sin and mental illness such as psychosis can be managed by church attendance. The questions were adapted from existing stigma scales, the depression self-stigma scale and the internalized stigma of mental illness scale^19,20^, and by incorporating acculturation and religious factors that are specific to Black immigrants. Our survey sought to capture specific stigmatizing views without being Eurocentric. In addition, both the Depression Self Stigma Scale (DSSS) and Internalized Stigma of Mental Illness (ISMI) scale assume the respondents have a mental illness, our survey was administered to measure perceived and experienced stigma without this assumption of known pathology as Black immigrants may be underdiagnosed due to low utilization of mental health services^21^. We administered this 37-question survey which included questions about depression, stress or anxiety, perceived stigma and experienced stigma, mechanisms to manage mental health and preferred use of resources. The full questionnaire and scoring instructions are available upon request from the corresponding author. The original Stigma and Culture Survey (SCS) captured salient themes previously identified in the current literature among Black immigrants.
3. The depression self-stigma scale (DSSS) is standardized and validated to measure stigma and its components (general self-stigma, secrecy, public stigma, treatment stigma, and stigmatizing experiences)^20^. DSSS scores ranged from 36–224 calculated as a sum with higher scores reflective of greater stigmatizing views.

### Study protocol

Twenty-three women responded to our invitation, one did not speak English and was excluded from the study, a total of 22 women participated in in-person surveys at the United African Organization and the Pan African Association after written informed consent was obtained on a voluntary basis. Each participant was compensated with $30 gift card for their time. The first author (ABP) supervised study sessions. Each survey took 15–30 minutes to complete. The study data was collected between January 2019 and June 2019 in 5 sessions. We addressed respondent social desirability bias by providing privacy during completion of the surveys.

### Statistical analysis

Total stigma score on the SCS was calculated by averaging responses to questions, “never” responses were coded as 1, “rarely” as 2, “sometimes” as 3, “very Often” as 4, and “always” as 5. Scores ranged from 1 to 5 with scores close to 5 indicating more stigma related to mental health. Overall stigma scores were broken down further into perceived and experienced stigma sub-scores. Calculation of DSSS scores followed standardized instructions^20^.

Descriptive statistics were used to summarize the cross-sectional sample of Black immigrant women. Frequency and percent were recorded for all categorical variables and both mean ± standard deviation, median, and range for continuous variables. Simple linear regression was used to determine the relationship between SCS and DSSS total score. Additionally, simple linear regression followed by overall (Type III Sum of Squares ANOVA) tests were used to determine whether an association existed between demographics (age group, marital status, gender, United States citizenship, education, employment status, socioeconomic status), health insurance, suicidality, depression, and spirituality/morality and either SCS overall stigma score or DSSS total score. All analyses were conducted using R (version 3.5.3, 2019, The R Foundation) and assumed a two-sided, 5% level of significance. We did not adjust for multiple hypothesis tests.

## Results

All participants identified as Black except for one who identified as Hispanic and one non-responder. While the majority of respondents recruited at the Pan African Association and United African Organization identified as Black immigrants, we did not exclude non-Black women. Fifty-four percent (N=12) were single or divorced, 45.5% (N=10) identified as African and 40.9% (N=9) identified as Black American. 40.9% (N=9) identified as United States citizens and 50% (N=11) identified as low-income. Complete socio-demographic data is presented in Table 1.

### Demographics and stigma scores

On average, United States citizens had lower stigma on the SCS [β 0.53; 95% CI: (0.94, −0.12); p-value: 0.02] and lower stigma on the DSSS [β 47.78; 95% CI: (67.97, −27.59); p-value: 0.0001] compared to non-US citizens. There was no significant association between individual components of socioeconomic status i.e. employment, education, income and stigma scores. We combined individual components of employment, education and income into socioeconomic status as a single variable and compared high versus low socioeconomic status. We found subjects with a higher SES had lower stigma scores for both scales compared to subjects with lower SES [SCS β: −0.65, 95% CI: (−1.26, −0.04), p-value: 0.0396; DSSS β: −41, 95% CI: (−79.16, −2.84), p-value: 0.0373]. We did not find a significant difference in stigma between middle and low socio-economic status, nor an association between stigma and other demographic variables examined (age and marital status).

### Association between overall DSSS and stigma and culture survey (SCS)

**Figure 1.**
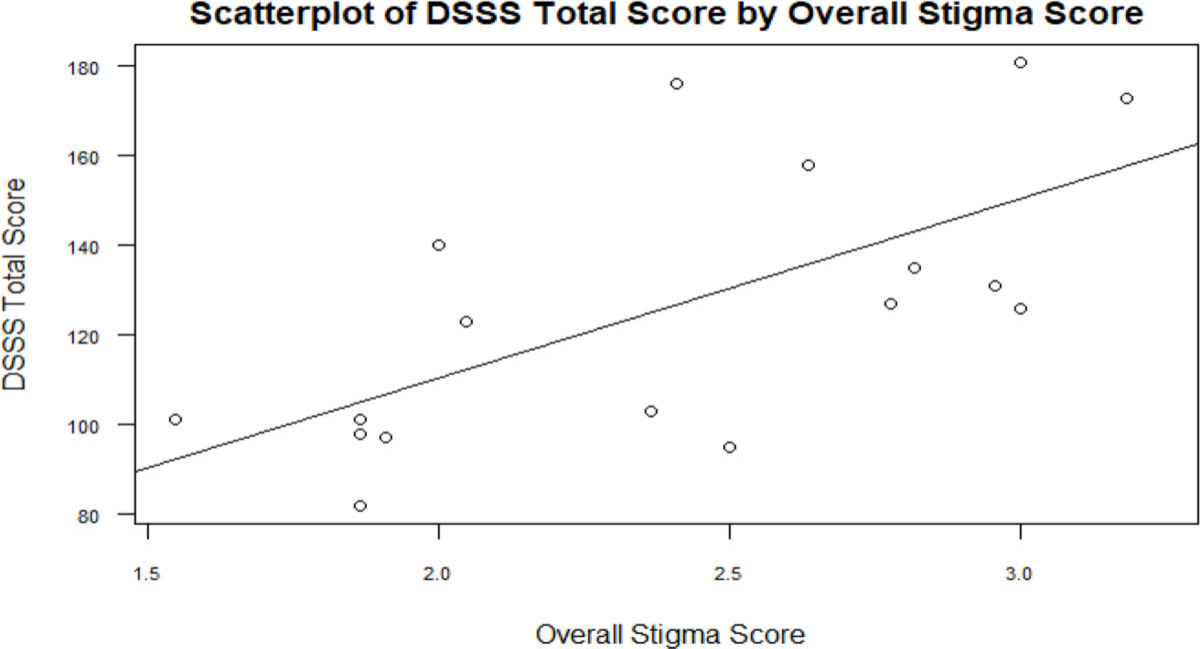
Relationship between the Depression Self Stigma Scale and the original Stigma and Culture Survey

A linear relationship was found between SCS overall score and DSSS total score. Specifically, a 1 point increase in SCS overall stigma score is associated with a 40 point increase in DSSS score on average [β: 39.95; 95% CI: (16.68, 63.21); p-value:0.004]. However, the overall stigma score explains 43% of the variability in DSSS overall score (see figure 1). The SCS and DSSS had a correlation coefficient of 0.66, suggesting a moderate to strong correlation. The two instruments had a linear relationship as shown in figure 1.

### Association between preferred use of resources, spirituality and stigma scores

Greater use of medical or biological resources (such as hospital, physician or medical resources) was associated with lower stigma scores among participants, as measured by the SCS [β: −0.3; 95% CI (−0.51, −0.09); p-value: 0.01]. Similarly, higher use of religious resources were associated with lower stigma scores for both the SCS [β: −0.38; 95% CI (−0.62, −0.15); p-value: 0.003] and the DSSS [β: −21.43; 95% CI: (−41.82, −1.04); p-value: 0.04]. On the other hand, higher levels of spirituality and morality (such as use of prayer, sinfulness attribution of mental illness) were associated with higher SCS stigma scores [β: 0.49; 95% CI: (0.19, 0.8); p-value: 0.003]. Spirituality and morality was associated with higher DSSS score [26.63; 95% CI (0.24, 53.02); p-value: 0.048].

## Discussion

In this pilot study, we explored the relationships between demographic factors and stigma scores among Black immigrant women. We used two stigma surveys: one was a validated and standardized survey, and the other was developed by the authors to additionally address specific cultural beliefs that may associated with stigma. These two surveys were found to be moderately to highly correlated. We found that spirituality was associated with higher stigma scores and use of religious resources was associated with lower stigma scores on both the SCS and DSSS measures. We define high spirituality as using activities like prayer to manage mental health needs and belief that mental illness is caused by sin or evil spirits. We define use of religious resources as those who would primarily seek counseling from a religious leader or church. Based on this pilot data, the use of religious resources did not confer higher stigmatizing views such that it is the very content of one’s beliefs that may confer higher stigmatizing views such as attribution of mental illness to sin or a self-inflicted moral failing. Hence we suggest that use of religious supports may have a positive impact on mental health but certain specific beliefs within one’s conceptualization of their faith may contribute to negative attitudes and possibly discriminatory behaviors towards oneself or towards others with mental illness^22,23^.

Respondents who identified as having United States citizenship had lower stigma scores on both instruments. Time spent in the United States may have an impact on levels of stigma. Existing literature has found that greater acculturation is associated with higher levels of mental health service use and greater ethnic identity is associated with lower levels of mental health service use^24^. While we did not assess generational status of those who were United States citizens, current research has called for taking into account immigrant and cultural background when assessing Black populations to better understand the content and characteristics of mental health stigma^3,12,15,16,25^.

Low socio-economic status correlated with higher stigma scores based on both the SCS and DSSS. Educational programming has been used to reduce mental illness stigma and is more effective among youth compared to adults^14^. It is unclear how employment, education and income individually affects mental health stigma among Black immigrants. We did not find individual components (such as employment, education and income) had a statistically significant association with stigma scores but we found an association when comparing these components as a single variable — socioeconomic status. In a large study addressing socioeconomic disparities among young adults (N>6000) in Europe, it was shown that those from low SES backgrounds had higher rates of unmet mental health needs^26^. Among those with severe mental illness such as schizophrenia, socioeconomic status influences mental illness outcomes^27^; however, the mechanism by which stigmatizing views moderate the relationship between socioeconomic status and mental health stigma outcomes remains unclear among Black immigrants.

Currently among stigma scales, there is a gap in stigma scales that have been standardized and validated among Black immigrants, we created a new measure to address this gap (SCS). Many existing scales focus on mental health service users rather than the general public and most of these commonly used scales have not been validated among Black immigrants^19,20^. Most stigma scales do not address the association of religion, spirituality and mental health stigma which are important factors to consider among Black immigrants as is highlighted in our study and consistent with the current literature^18,28^. Spirituality and religion play a significant role in the understanding of mental illness among the general public^29^, and in particular among immigrants^30,31^. We have designed a survey that was compared to the validated and standardized depression self-stigma scale and found a positive correlation between our survey which considers the association of mental health stigma, religion, culture and spirituality in assessment of stigmatizing views. Our survey incorporated factors such as citizenship status and ethnicity. Among immigrants, the role of migration and the incidence of mental illness is an area of ongoing investigation, for example migration confers an increased risk of severe mental illness among first and second generation immigrants^32,33^.

The World Health Organization and the World Psychiatric Association have proposed that religion and spirituality are integral to comprehensive mental health care, citing that for many service users, in order to provide culturally competent care, addressing religion and spirituality is essential^22,34^. Among a random sampling of 1144 physicians, psychiatrists were found to be less religious than other physicians, making it a challenge for some psychiatrists to be empathic towards their religious patients^35,36^. This pilot study suggests that religion and spirituality have key associations with mental illness stigma. The relationship among mental health stigma, religion and spirituality is important when addressing Black immigrant mental health.

### Critique and Future direction

We collected surveys from a group of Black immigrant women who were members of two community based Black immigrant organizations. Black immigrants predominantly have their origin in African and Afro-Caribbean countries. While Black immigrant women do not represent a homogeneous group, our study considers the influence of migration among Black people by focusing on Black immigrant women in assessing mental illness stigma characteristics. The small sample size limited assessment of within group variations, though we present on several factors that were found to be correlated with stigmatization, thus laying a foundation for future studies. This was an exploratory study, we plan to conduct a larger study that will further examine the association between religion, culture and stigma among Black immigrants and further assess the diversity within this population. We identified several cultural and religious factors associated with mental illness stigma. Our study was conducted in English, future studies assessing stigma among Black immigrants would benefit from including more languages as spoken across African and Afro-Caribbean countries. Some definitions in our study have multiple meanings; for example, religion and spirituality do not have definitive definitions in the community. We addressed this by clarifying what we were measuring in our survey, for example use of religious resources such as a church leader versus religiosity, which is defined variably in the academic community and among laypersons.

## Conclusion

Our study sought to examine how mental health stigma is associated with mood symptoms, religion and spirituality among Black immigrants. We found citizenship, socioeconomic status and religious frameworks were associated with stigmatizing views among Black immigrant women. We hope our findings will inform future research especially as it relates to building stigma reducing interventions that are efficacious and effective among marginalized populations including among Black immigrants in the United States.

## Figures and Tables

**Table 1.** Participant characteristics

	Overall (n=22)
**Age Group**	
18–24	8 (36.4%)
25–40	7 (31.8%)
41–65	6 (27.3%)
Non-responder	1 (4.5%)
**Marital Status**	
Married/Partnered	9 (40.9%)
Single/Divorced	12 (54.5%)
Non-responder	1 (4.5%)
**Gender**	
Female	22 (100%)
**Ethnicity**	
African	10 (45.5%)
Afro-Caribbean	1 (4.5%)
Black/African American	9 (40.9%)
Other Hispanic	1 (4.5%)
Non-responder	1 (4.5%)
**Level of Education**	
High School/GED or Less	7 (31.8%)
Some College	8 (36.4%)
Bachelor's Degree or Above	7 (31.8%)
**Source of Income**	
Family/Friends	7 (31.8%)
Government	1 (4.5%)
Other	6 (27.3%)
Self	8 (36.4%)
**Employment Status**	
No	10 (45.5%)
Yes	11 (50.0%)
Non-responder	1 (4.5%)
**Income**	
Less than $20,000	13 (59.1%)
More than $20,000	6 (27.3%)
Non-responder	3 (13.6%)
**Are you a US citizen?**	
No	11 (50.0%)
Yes	9 (40.9%)
Non-responder	2 (9.1%)
**Socioeconomic Status**	
Low	11 (50.0%)
Middle	5 (22.7%)
High	3 (13.6%)
Non-responder	3 (13.6%)

**Table 2.** Simple Linear Regression Results: Evaluating the Association between Participant Characteristics and Mental Health Stigma (N=22)^1^

Variables	SCS : Overall Estimate (95% CI); p-value	DSSS: Total Estimate (95% CI); p-value
Age Group 25–40 vs 18–24	0.1 (−0.49,0.69); 0.72	−9.12 (−49.26,31.01); 0.63
41–65 vs 18–24	0.09 (−0.52,0.71); 0.75	−0.62 (−43.74,42.49); 0.98
Marital Status Single/Divorced vs. Married/Partnered	0.14 (−0.33,0.61); 0.54	9.19 (−26.23,44.61); 0.59
US Citizenship (Yes vs No)*	−0.53 (−0.94, −0.12); 0.01*	−47.78 (−67.97, −27.59); 0.00*
Some College vs High School/GED or Less	−0.33 (−0.88,0.21); 0.21	16.03 (−24.19,56.24); 0.41
Bachelor’s Degree or Above vs High School/GED or Less	−0.02 (−0.58,0.54); 0.95	18 (−25.44,61.44); 0.39
Income More than $20,000 vs Less than $20,000	−0.35 (−0.85,0.16); 0.17	−15.6 (−50.61,19.41); 0.35
Employment (Yes vs No)	0.05 (−0.44,0.53); 0.84	−17.39 (−49.13,14.35); 0.26
Socio-economic Status* Middle vs Low	0.17 (−0.34,0.68); 0.48	−3 (−41.16,35.16); 0.87
High vs Low*	−0.65 (−1.26, −0.04); 0.04*	−41 (−79.16, −2.84); 0.04*
Religious Resources*	−0.38 (−0.62, −0.15); 0.00*	−21.43 (−41.82, −1.04); 0.04*
Community, Family, Friend Resources	−0.18 (−0.47,0.12); 0.23	−4.7 (−25.38,15.98); 0.63
Biological, Formal, Medical Resources*	−0.3 (−0.51, −0.09); 0.01*	−2.04 (−18.25,14.18); 0.79*
Spirituality/Morality*	0.49 (0.19,0.8); 0.00*	26.63 (0.24,53.02); <0.05*

1 Estimates of association between stigma (SCS and DSSS) and demographic factors, citizenship, socioeconomic status, religiosity and use of resources among women at two Black immigrant community centers in Chicago (N=22) are provided. Coefficient or β estimates from simple linear regression and corresponding 95% confidence interval (CI) are reported. P-values are obtained from t-tests from the regression model.
